# What Do I Want and When Do I Want It: Brain Correlates of Decisions Made for Self and Other

**DOI:** 10.1371/journal.pone.0073531

**Published:** 2013-08-22

**Authors:** Konstanze Albrecht, Kirsten G. Volz, Matthias Sutter, D. Yves von Cramon

**Affiliations:** 1 Institute of Psychology, RWTH Aachen University, Aachen, Germany; 2 Werner Reichardt Centre for Integrative Neuroscience, University of Tübingen, Tübingen, Germany; 3 Department of Public Finance, University of Innsbruck, Innsbruck, Austria; 4 Department of Economics, University of Cologne, Cologne, Germany; 5 Max Planck Institute for Neurological Research, Cologne, Germany; 6 Department of Cognitive Neurology, Max Planck Institute for Human Cognitive and Brain Sciences, Leipzig, Germany; University of Bologna, Italy

## Abstract

A number of recent functional Magnetic Resonance Imaging (fMRI) studies on intertemporal choice behavior have demonstrated that so-called emotion- and reward-related brain areas are preferentially activated by decisions involving immediately available (but smaller) rewards as compared to (larger) delayed rewards. This pattern of activation was not seen, however, when intertemporal choices were made for another (unknown) individual, which speaks to that activation having been triggered by self-relatedness. In the present fMRI study, we investigated the brain correlates of individuals who passively *observed* intertemporal choices being made either for themselves or for an unknown person. We found higher activation within the ventral striatum, medial prefrontal and orbitofrontal cortex, pregenual anterior cingulate cortex, and posterior cingulate cortex when an immediate reward was possible for the observer herself, which is in line with findings from studies in which individuals actively *chose* immediately available rewards. Additionally, activation in the dorsal anterior cingulate cortex, posterior cingulate cortex, and precuneus was higher for choices that included immediate options than for choices that offered only delayed options, irrespective of who was to be the beneficiary. These results indicate that (1) the activations found in active intertemporal decision making are also present when the same decisions are merely observed, thus supporting the assumption that a robust brain network is engaged in immediate gratification; and (2) with immediate rewards, certain brain areas are activated irrespective of whether the observer or another person is the beneficiary of a decision, suggesting that immediacy plays a more general role for neural activation. An explorative analysis of participants’ brain activation corresponding to chosen rewards, further indicates that activation in the aforementioned brain areas depends on the mere presence, availability, or actual reception of immediate rewards.

## Introduction

If asked to choose between a smaller but sooner monetary reward and a larger but later monetary reward, people more often prefer the smaller reward if it is available immediately than if it is available only after some delay [1,2,3]. Such a choice pattern -- documented in a great number of psychological and economic studies on intertemporal choice behavior [e.g. 4,5] -- leads to dynamically inconsistent behavior. While, for example, a person prefers receiving €10 today to receiving €12 in two weeks, she might not prefer receiving €10 in one week to receiving €12 in three weeks, even though the monetary and temporal difference is the same in both cases (€2 and two weeks, respectively). This finding has been interpreted to reflect individuals’ impatience in the face of immediate gratification (i.e., a reward with no delay at all), and their patience when confronted with choice sets involving delayed rewards only [e.g. 1, 2, 6]. To explain such inconsistent choices -- which violate the axioms of microeconomic theory defining economically rational behavior [7] -- researchers often draw on a dual-system framework [8,9,10,11,12,13]. According to such a separate systems hypothesis, time-inconsistent preferences are assumed to be caused by the activity of two separate systems, one pertaining to impulsiveness (i.e., immediate gratification), and the other pertaining to self-control. (Yet the suggestion of separate systems being responsible for impulsive and pondered decisions has not been left unchallenged [e.g. 14, for a more general critique of dual-systems models see 15,16].)

McClure and colleagues [4,10] identified a network of brain areas involved in intertemporal choice; activation of reward- and emotion-related areas, such as the ventral medial prefrontal cortex (vMPFC), pregenual anterior cingulate cortex (pACC), the posterior cingulate cortex (PCC), and the ventral striatum (vStr) was higher when immediate gratification was possible than when only delayed rewards were involved. McClure et al. interpreted this activation pattern within the construct of the proposed dual-system framework [9,10,11,12,13], suggesting that immediate rewards trigger activation in the network pertaining to impulsiveness more than delayed rewards do.

In a previous study, we investigated the effect of personal involvement on intertemporal choice in order to determine the extent to which intertemporal choices for another person are accompanied by the same brain activation patterns as choices for the self [17]. We replicated the findings by McClure and colleagues [10], but discovered that the findings are dependent on personal involvement: When participants made decisions for themselves, a difference in activation occurred between choices involving an immediate option (“today trials”) and choices involving only delayed options (“delay trials”) in the MPFC, pACC, and ventral striatum. In contrast, we observed no activation differences between today trials and delay trials when intertemporal decisions were made for an unknown person. These results suggest that who benefits from the choice (i.e., me or another person) is important, and that personal involvement specifically affects the perception of immediacy of rewards. These results can also be taken to support the assumption that choices made for someone else are characterized by more patience. This finding is in keeping with Parfit’s “multiple selves over time” approach: He argues that the “self” changes over time, and that future selves (e.g., me in two weeks) are probably considered more similar to others (e.g., another person *now*) than to one’s present self [cf. 18,19]. Similarly, a study by Hershfield and colleagues [20] showed that neural measures of self-continuity (i.e., how similar one’s present self is perceived to be to a future self) correspond to intertemporal choice behavior: Individuals who showed similar brain activation when judging to what extent a trait adjective described them both now and in the future chose more patiently than individuals who showed a larger activation difference when judging their future versus their present selves [cf. 18,20].

In the present study, we examined the effect of personal involvement on intertemporal choice from a different angle. We investigated how activation patterns and cognitive processing change when participants do not make intertemporal decisions themselves but are only informed of the options while another person makes the actual choices for them. The personal involvement is maintained in the sense that it is *me* getting a smaller amount of money now or a larger amount of money later, yet the actuation of intertemporal choice is under the control of someone else who is effectively choosing which sort of reward I get. Such situations are common in everyday life -- for example, when professionals make investment decisions for their clients, or when parents decide for their children. There might be a good reason to shift the power of decision to induce more patient and less impulsive choices. Hence, we study whether the brain activations found in active intertemporal decision making are also present when the same decisions are merely observed. An affirmative answer would support the idea that a robust brain network is engaged whenever immediate gratification is available, irrespective of whether decisions are actively made or passively observed.

In addition, we varied personal involvement by having as the beneficiary of a decision either the person observing the intertemporal choice herself, or another, unknown person. Based on our results in the previous study [17], we wanted to investigate whether the differing activation patterns that have been found to exist in situations where individuals make intertemporal choices for themselves and situations where the beneficiary is another person also occur when those individuals are merely *observing* the intertemporal choices being made for them and for others. If these patterns do occur, it would imply that brain activity differentiates with respect to the beneficiary of intertemporal choice even when active control is not possible. Thus in the present study we investigate whether previous findings on reward-related activation also prevail in a new setting in which preferences cannot be determined behaviorally.

We expect increased activation in the aforementioned emotion- and reward-related areas (ventral Striatum, MPFC, MOFC, pACC, and PCC) when immediate personal gratification for the self is an option, which would be consistent with studies on observational learning [21] as well as experiments investigating brain correlates of valuation in the absence of choice [22].

This assumption should be reflected in the interaction of “beneficiary of the choice” (receiver type: SELF vs. OTHER) and “immediacy” (temporal distance: today vs. delay) as has been shown in Albrecht et al. [17] within an active decision context: Activation in the aforementioned areas should be higher for the combination of SELF and today than for all other combinations of receiver type and temporal distance.

## Methods

### Participants

Thirty right-handed, healthy volunteers (15 females) were recruited to participate in the study (mean age 25.1 years; SD: 2.9; range 20-31). All participants gave informed written consent before participating. The experimental standards were approved by the local ethics committee of the University of Leipzig. Data were handled anonymously. Before the experiment, participants were instructed that the experimental session consisted of two parts, and that the instructions for an as yet unexplained second part would be presented on the display in the fMRI-scanner after the first part was completed.

### Behavioral task and stimuli

The design of the study was adapted from McClure et al. [10]. Participants observed intertemporal decisions that were made by another person referred to as the “decision maker.” The decision maker was a confederate, but was introduced to each participant as another participant. To keep potential influence factors constant, we used the same person as the decision maker throughout the experiment. In part one of the experiment, half of our participants were exposed to the SELF-condition, with the decision maker making choices for the observing participant, while the other half were exposed to the OTHER-condition, with the decision maker making choices for a second unknown person while they observed. In part two of the experiment, the SELF and OTHER conditions were reversed (see Table 1).

**Table 1:** Overview of conditions.

	SELF		OTHER	
	Today trials	Delay trials	Today trials	Delay trials
Number of experimental trials	16	24	16	24
Immediate gratification	Possible	Not possible	Possible (for another person)	Not possible (for another person)
Number of catch trials	10		10	

Participants never met the second, unknown person. They knew only that the person was another participant. To make this second person more concrete and to make it clear that the person and the decision maker were not in fact the same, we displayed on a screen 15 photographs of individuals (aged 18-30 years) and told our participants that one of these individuals would be the other person for whom choices were made by the decision maker [23]. Only photographs with average attractiveness ratings were presented to our participants. Attractiveness ratings were elicited beforehand, by a different sample.

Participants faced the same choice options as those in our earlier study [17]. However, in the present study participants did not choose for themselves at all but observed choices being made for them (or for that other, unknown person) by the decision maker. To make the observed choices realistic and comparable to the earlier study, we used the choice frequencies for the immediate option from the earlier study and implemented them in the following way: If, for a given choice set, participants in the previous study chose the immediate option in X% of all cases, the immediate option was also implemented in exactly X% of choices in the experiment presented here, i.e., the decision maker chose the immediate option for the participant being scanned in X% of all cases. Please see supporting information file S1 for the exact instructions given to the participants.

In each part of the experiment, participants observed 40 choices being made by the decision maker between a smaller, sooner reward and a larger, later reward. In order to maintain participants’ attention and control for attention differences between the conditions of SELF and OTHER, we randomly inserted a total of ten catch trials in each part of the experiment (with the restriction that there could not be two catch trials in a row) where participants were required to make an intertemporal choice themselves. Participants were instructed to make a choice (i.e., to press the button indicating their preferred option) whenever the delay (in weeks) to the reward equaled the amount of the reward in euros (e.g., €10 in 10 weeks or €12 in 12 weeks). Participants were aware that choices in these catch trials would not be paid out and were not visible to the decision maker (who would of course be outside of the scanner). These trials were used to make sure that participants paid attention. All catch trials are listed in the supporting information file S1 (Tables S1 and S2).

**Figure 1:**
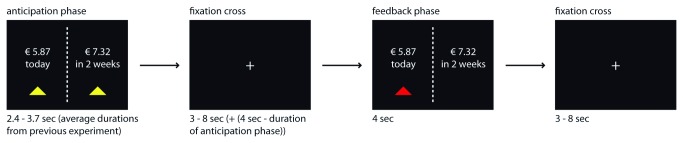
Paradigm: Choices were presented on a screen with the smaller, sooner reward always presented on the left side. After the subsequent presentation of a fixation cross (inter-trial interval), participants received feedback about the choice that was made by the decision maker.

All choices were presented on a screen with the smaller, sooner reward always presented on the left side (Figure 1). The duration of the presentation of the choice options varied between 2.6 and 3.4 seconds, matching the average amount of time that participants in our previous study took to make the respective choice [17]. Subsequently, a fixation cross was presented in the center of the black screen, which jittered for 3 to 9 seconds and was followed by a feedback phase in which the (previous) intertemporal choice was presented with the selected option highlighted for 4 seconds. The jittered presentation of the fixation cross served as the inter-trial interval. Feedback was given in order to show participants that a choice was actually made for them or for another person. Accordingly, the feedback phase was modeled in the general linear model (GLM). So that participants would have the impression that they had no influence on the decision maker, they could not indicate during the experiment how satisfied they were with the decision maker’s choices. Thus the feedback phase included a factor that hinders a clear interpretation: We could not distinguish between feedback trials in which participants received a desired feedback (e.g. the sooner option was chosen for and preferred by them) and those in which participants received an undesired feedback (e.g., the sooner option was chosen, but they would have preferred the later option). Despite this potential confound, we consider the insights that the results of the feedback phase could give us as relevant, because they can shed light on whether the mere presence, the availability, or the actual reception of immediate rewards drives the activation differences in the respective brain areas. Therefore we will report results from an explorative analysis of this feedback phase and discuss its effects.

In the ten catch trials, participants were allowed 10 seconds to respond. After indicating their choice by pressing one of two buttons spatially corresponding to the two stimulus locations, a feedback confirmed their respective choice. Subsequently, a fixation cross was presented and the next trial started.

The 40 choice situations in the experimental trials always included a sooner, smaller reward (r1), and a larger, later reward (r1’). Rewards r1 were randomly drawn from a Gaussian distribution (mean: €20; standard deviation: €10; minimum: €5; maximum: €40). The corresponding later, larger rewards (r1’) were calculated by adding a percentage (x) of either 1, 3, 5, 10, 15, 25, 35 or 50 to the sooner reward. The assignments of percentages to the sooner rewards were quasi random so that every percentage rate was used five times. The sooner rewards were available at the following times (t): “today,” in “two weeks,” and “in four weeks.” The span of time (t'-t) between the sooner and the later reward was either two weeks or four weeks. All combinations of sooner rewards and delays were used eight times each, except for the combination of “in four weeks” and “in eight weeks,” which was not used. Each combination was then assigned to all available percentage rates and thus quasi randomly to the rewards connected to those rates. Since the experiment consisted of two parts, the whole procedure was repeated with another forty, sooner, smaller rewards (r2) that were randomly drawn from the Gaussian distribution mentioned above. The presentation order of the choice sets (r1/r1’ and r2/r2’) was balanced across parts, and the presentation order within the choice sets was balanced across participants.

As outlined above, the decisions made by the decision maker were not randomly chosen or otherwise artificially determined but corresponded to the choice frequencies for a specific trial determined from participants’ choice behavior in an earlier study for the same intertemporal choice options [17]. In trials containing an immediate option, that option was chosen in 51% of such cases. In trials with only delayed options, the sooner option was chosen in 41% of these cases. For an overview of all rewards and delays to payment and the actual choice communicated to observers, see supporting information file S1, Tables S1 and S2.

Each part of the experiment took about 15 minutes; the experiment took about 30 minutes total. At the end of the fMRI experiment, the reward from one trial from each of the two parts of the experiment was randomly selected and paid out to the participants at the corresponding point in time. The reward chosen in the SELF-condition was delivered in cash to the observer on the date indicated in the chosen option. Immediate rewards were paid directly after the experiment; delayed rewards were delivered to the observer’s home or a more convenient place determined by the observer. In addition to the earnings from the SELF-condition, every participant was paid an additional €8 for taking part in the experiment. Catch trials were not paid because we wanted to avoid participants’ having an influence on the monetary outcome; we were interested in observation effects only.

### Imaging

Imaging was performed on a 3 Tesla scanner (Siemens TRIÓ, Erlangen, Germany); 26 axial slices (4mm thickness, 20% spacing, field of view [FOV)] 19.2cm, data matrix of 64x64 voxels, and in-plane resolution of 3mm x 3mm) parallel to the bi-commissural plane (AC-PC) covering the whole brain were acquired using a single-shot echo-planar imaging (EPI) sequence (TR 2s, echo time [TE] 30ms, flip angle 90°). Two functional runs with 465 time points each were run with each time point sampling over the 26 slices. Prior to functional runs, 26 anatomical T1-weighted modified driven equilibrium Fourier transform (MDEFT) images (data matrix 256x256, TR 1.3s, TE 10ms) were acquired [24,25] as well as 26 T1-weighted EPI images with the same spatial orientation as the functional data. The latter were used to co-register the functional scans with previously acquired high-resolution full-brain 3D brain scans. (Data is available upon request. Please contact the corresponding author at albrecht@psych.rwth-aachen.de.)

### *Data* analysis:

The MRI data were processed using the software package LIPSIA [26]. Functional data were motion-corrected offline with the Siemens motion correction protocol (Siemens, Erlangen, Germany). To correct for the temporal offset between the slices acquired in one scan, a cubic-spline-interpolation was applied. A temporal highpass filter with a cut-off frequency of 1/120 Hz was used for baseline correction of the signal, and a spatial Gaussian filter with 5.65mm full width half-maximum (FWHM) was applied. The anatomical slices were co-registered with the high-resolution full-brain scan that resided in the stereotactic coordinate system and then transformed by linear scaling to a standard size [27]. The transformation parameters obtained from this step were subsequently applied to the preprocessed functional slices so that the functional slices were also registered into the stereotactic space. This linear normalization process was improved by an additional nonlinear normalization known as “demon matching”. An anatomical 3D dataset (i.e., the model) is “deformed” so that it matches another 3D anatomical data set (i.e., the source) that serves as a fixed reference image [28]. This 3D reference dataset was acquired for each participant during a previous scanning session. The MDEFT volume data set with 160 slices and 1mm slice thickness was standardized to the Talairach stereotactic space [27]. The voxel size was interpolated during the co-registration from 3mm x 3mm x 4mm to 3mm x 3mm x 3mm. The statistical evaluation was based on a least-squares estimation using the general linear model (GLM) for serially autocorrelated observations [random effects model; 29,30,31]. The general linear regression performs a “prewhitening” of the data, i.e., the autocorrelation parameters were used to “whiten” the data and the design matrix. Then the linear model was reestimated using least squares on the whitened data to produce estimates of effects and their standard errors.

The design matrix included the following conditions: T (today trials, i.e., trials containing an immediate and a delayed option), D (delay trials, i.e., trials in which the sooner reward is delayed by at least two weeks), FBT (feedback in today trials), FBD (feedback in delay trials), NULL (baseline periods before and after T and D) and C (catch trials including response feedback). The duration of T and D varied from 2.6 to 3.4 seconds. The duration of FBT/FBD was always 4 seconds, while the duration of NULL varied from 3.6 to 9.4 seconds (providing even jittering). Conditions were modeled separately for SELF and OTHER, and then concatenated.

An event-related design was used. The design matrix was generated with a synthetic hemodynamic response function [32,33]. The model equation, including the observation data, the design matrix, and the error term, was convolved with a Gaussian kernel of dispersion of 4 seconds FWHM to deal with the temporal autocorrelation [31].

Contrast images (i.e., estimates of the raw-score differences between specified conditions) were generated for each participant. The single subject contrast images were entered into a second-level analysis on the basis of Bayesian statistics [see also 34,35]. In the approach by Neumann and Lohmann [35], posterior probability maps and maps of the effect size are calculated on the basis of the resulting least-squares estimates of parameters for the GLM. The output of the Bayesian second-level analysis is a probability map showing the probability for the contrast to be larger than zero. This Bayesian technique allows us to directly estimate the probability of a specific difference in the group means given the parameter estimates of the GLM for the individual participants. This is more informative than a classical rejection of a null hypothesis. This approach has the further advantage of being less sensitive to outliers than traditional t-statistics, as the influence of individual participants on a group statistic is weighted by the within-subject variability. In support of this, Thirion et al. [36] recently suggested that, from the point of view of reliability, optimal statistical thresholds for activation maps are lower than classical thresholds corrected for multiple comparisons. Furthermore, since probabilities of the contrasts are calculated, but no significance tests are performed, corrections for multiple comparisons or calculations of effect sizes are not necessary. For visualization, a threshold of 99% was applied to the probability maps. Additionally, for all voxels of a region of interest (ROI), a contrast value (i.e., parameter estimate from the GLM) was generated for each contrast and participant. Clusters from the interaction contrast ([T_SELF_-D_SELF_]-[T_OTHER_-D_OTHER_]) during the presentation of the choice options were used to define these ROIs. Parameter estimates are displayed, for illustration purposes only, to visualize differences that were detected in a whole brain analysis in the interaction contrast.

## Results

We assumed that potential differences in the level of attention between the conditions SELF and OTHER would be reflected in different reaction times in catch trials. To this end, we used a paired-samples t-test to test for differences between mean catch-trial response times for the two conditions. We did not find a difference, which seems to suggest equally sustained attention (*t*(29)=-0.932*, p*=.359). We further tested response time differences in catch trials *following* today and delay trials to compare attention in today trials and delay trials. A paired-samples t-test yielded no differences in mean catch-trial response times (*t*(29)=0.465*, p*=.649). These results indicate equally sustained attention in today and delay trials. Reaction times of the catch trials are displayed in Table S3 in the supporting information file S1.

### Activation differences during the presentation of the choice options

To test for the specific brain correlates of choices containing an immediate reward option (today trials), we contrasted the hemodynamic response elicited by this kind of trial with those decision trials containing only delayed options (delay trials). We calculated separate contrasts for the two receiver types (SELF and OTHER), as well as an interaction contrast of temporal distance (today vs. delay trials) and receiver type (SELF vs. OTHER) for the presentation of the choice options.

**Figure 2:**
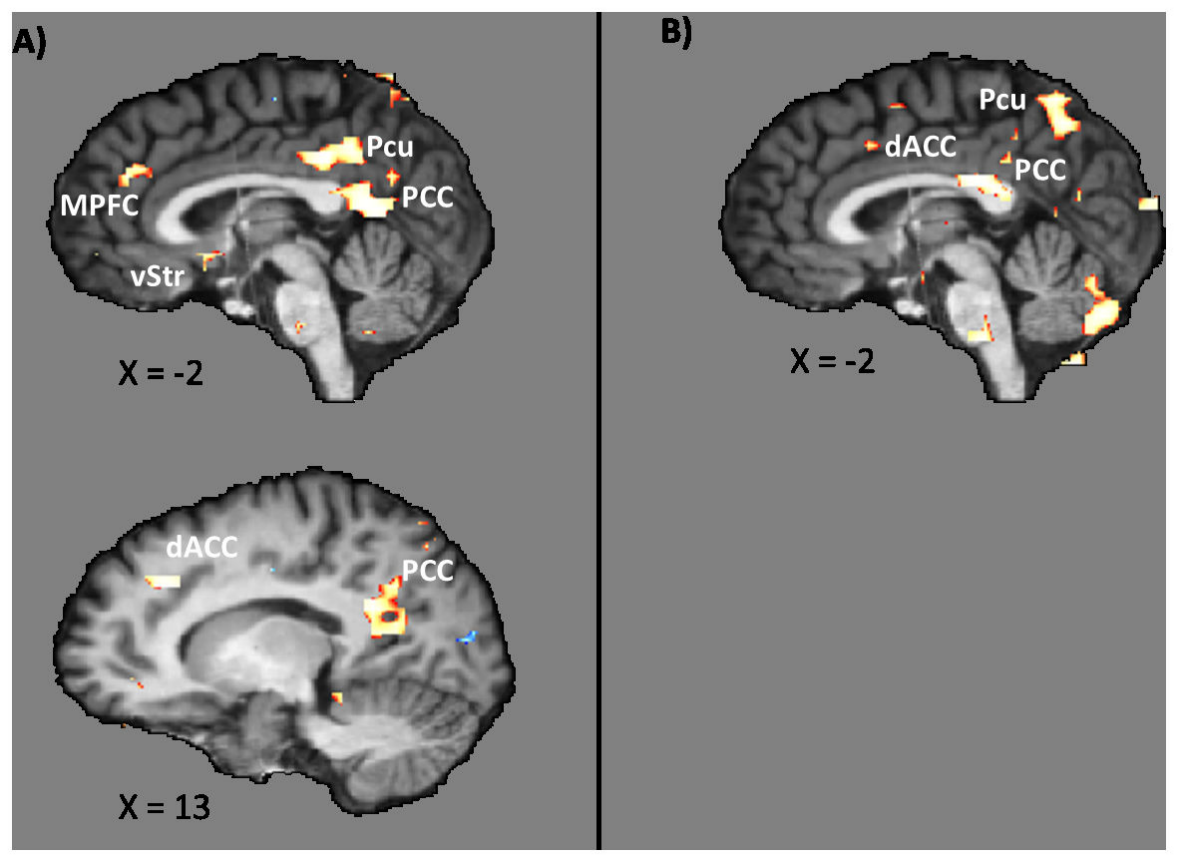
Contrasts of today trials with delay trials in A) SELF, and B) OTHER. Abbreviations: dACC: dorsal anterior cingulate cortex, MPFC: medial prefrontal cortex, PCC: posterior cingulate cortex, Pcu: precuneus, vStr: ventral striatum.

The main contrast of today trials vs. delay trials in SELF yielded higher activation for today trials within the ventral striatum (vStr), medial orbitofrontal cortex (MOFC), medial prefrontal cortex (MPFC), dorsal anterior cingulate cortex (dACC), ventral and dorsal posterior cingulate cortex (vPCC, dPCC), and precuneus (Pcu) (see Figure 2A, cf. supporting information file S1, Table S4). In the OTHER condition, significantly higher activation was found for today trials solely within the dACC, PCC, and Pcu. Activation within the vStr, MOFC, or MPFC was not found with the threshold of 99% (see Figure 2B, cf. supporting information file S1, Table S4).

**Figure 3:**
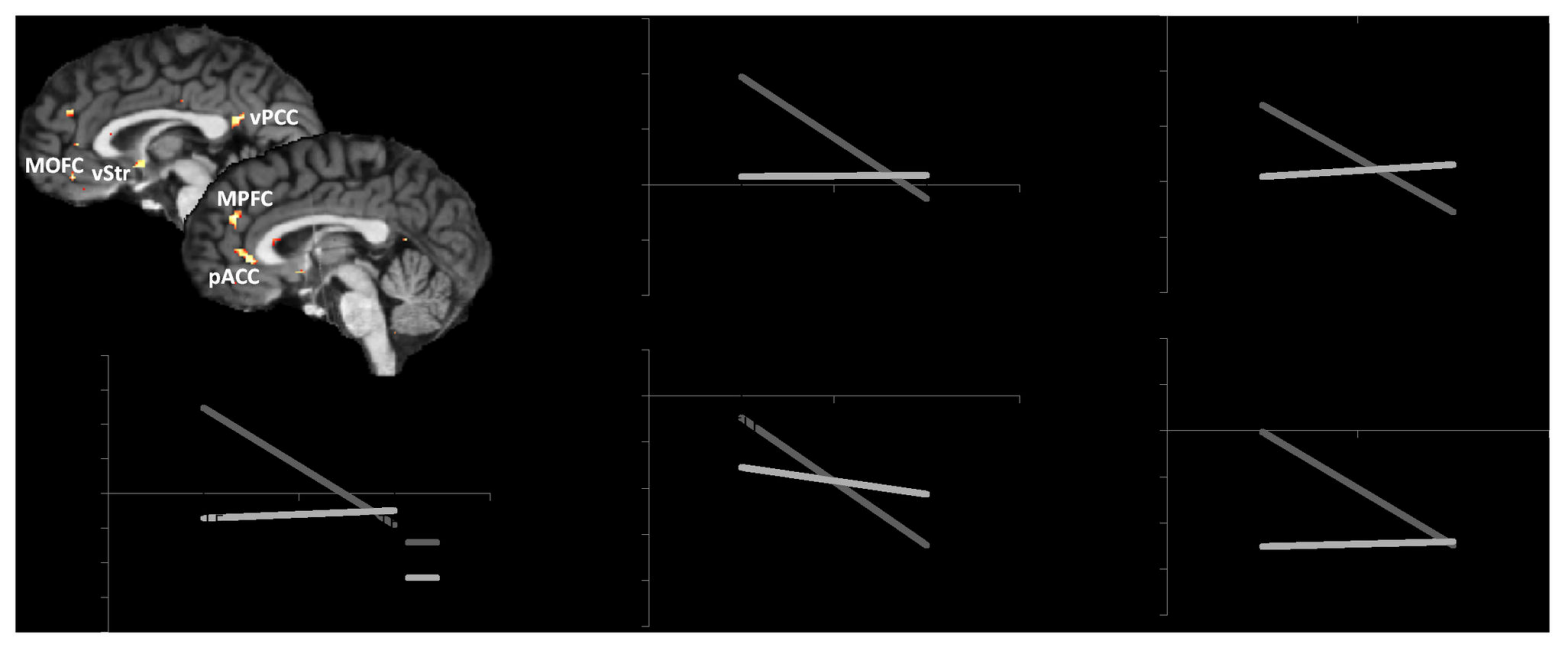
Interaction contrast of receiver and temporal distance (SELF [today-delay] – OTHER [today-delay]). Parameter estimates (including standard error) are displayed for illustration of the interaction effects only. Abbreviations: MOFC: medial orbitofrontal cortex; MPFC: medial prefrontal cortex; pACC: pregenual anterior cingulate cortex; vPCC: ventral posterior cingulate cortex; vStr: ventral striatum.

An interaction contrast of receiver type (SELF vs. OTHER) and temporal distance (today vs. delay) yielded significant differences in the vStr, MOFC, MPFC, pACC, and vPCC (see Figure 3, cf. supporting information file S1, Table S4). Together with the results from our main contrasts (see Figure 2), these findings support the assumption that activation was higher only when the participant herself was the receiver of an immediate reward compared to a delayed reward. Parameter estimates drawn from these areas further support this and suggest that activation was even highest when the participant herself was the receiver of an immediate reward compared to all other combinations of receiver type and temporal distance (see Figure 3, cf. supporting information file S1, Table S4). The main effect of receiver type (SELF vs. OTHER, not split for temporal distance) is displayed in Table S4. This contrast revealed significant activation within the pACC, dACC, and Cuneus. Such a pattern of activation is in line with the literature on differences between stimuli processing concerning oneself and stimuli processing concerning others, which is suggested to reflect self-related processes [37,38]; (cf. supporting information file S1, Table S4). Since this result is not a part of our main analysis, it is not discussed further.

### Activation differences during the feedback phase

To keep participants’ impression of their influence on the decision maker as low as possible, they could not indicate during the experiment how satisfied they were with the choices made by the decision maker. Hence, we could not distinguish between brain activation accompanying desired versus undesired choice outcomes. Yet, we consider the insights that activation differences during the feedback phase may reveal as relevant nevertheless and will report the results now, acknowledging that an interpretation has to be made with caution. We would like to thank an anonymous reviewer for suggesting this analysis.

**Figure 4:**
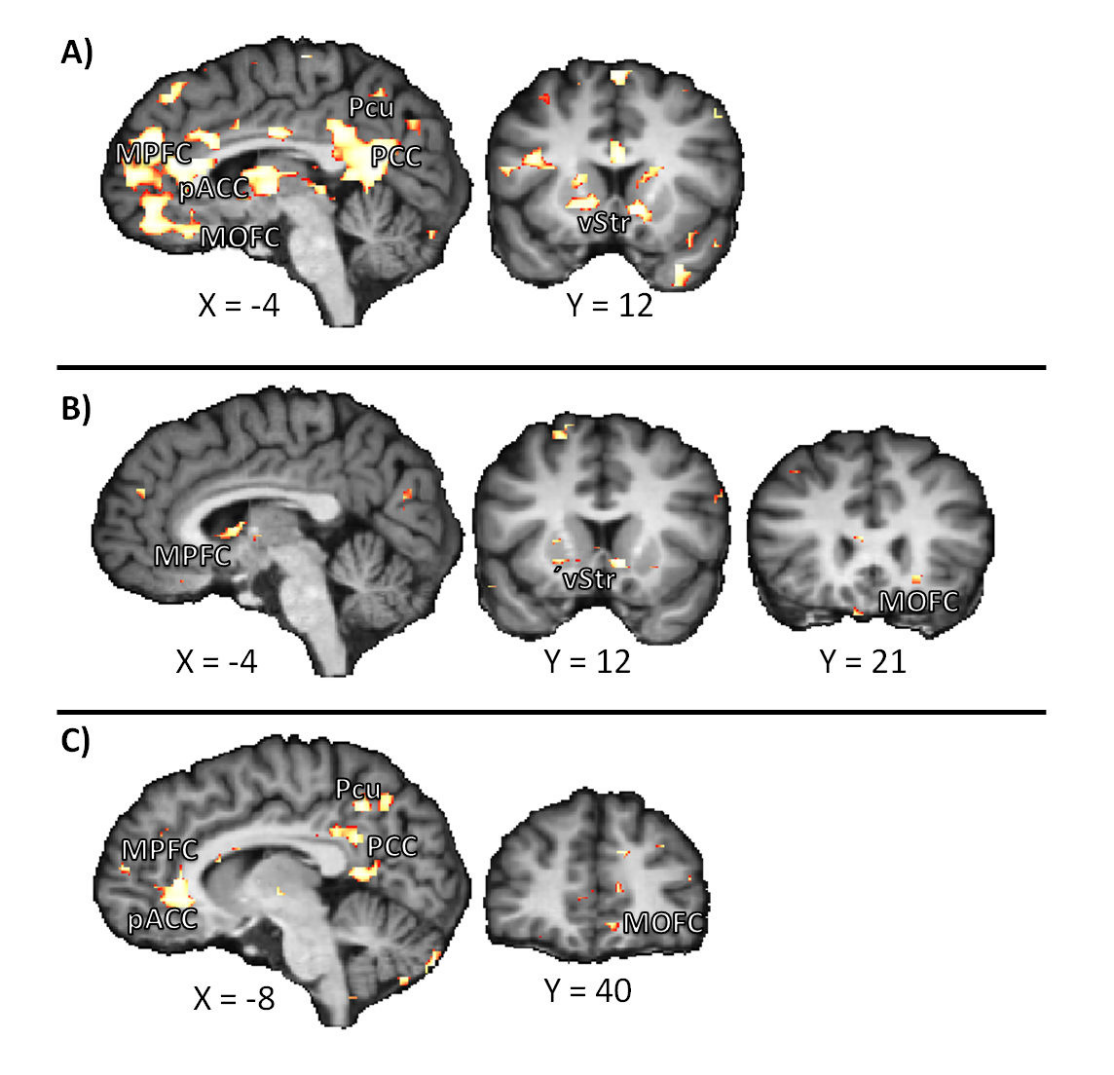
Response-dependent contrasts of the feedback phase. We contrasted **A**) the choice of the sooner option in today trials with the choice of the sooner option in delay trials, **B**) the choice of the sooner reward in today trials with the choice of the later reward in today trials, and **C**) the choice of the later reward in today trials with the choice of the later reward in delay trials. Abbreviations: MPFC: medial prefrontal cortex, MOFC: medial orbitofrontal cortex, pACC: pregenual anterior cingulate cortex, PCC: posterior cingulate cortex, Pcu: precuneus, vStr: ventral striatum.

We were interested in the question whether the increased activation in the reward- and emotion-related brain areas we found in today trials for SELF during the presentation of the choice options, would still be present after a choice in favor of the immediate option was made. To this end, we calculated the following response-dependent contrast in the SELF condition: We contrasted choices for today in today trials with choices for the early option in delay trials. Activation was higher in the vStr, MOFC, MPFC, pACC, PCC, and Pcu when today was chosen in the today trials than when the early option was chosen in delay trials (see Figure 4A, cf. supporting information file S1, Table S5). To further investigate our question, we calculated the following response-dependent contrast for today trials (in the SELF condition): We contrasted choices for today (i.e., immediate gratification) with choices for delay in today trials. This contrast yielded higher activation in the vStr, MOFC, and MPFC when today was chosen as compared to when delay was chosen in today trials (see Figure 4B, cf. supporting information file, Table S5).

Lastly, we investigated whether the presence of an immediate reward per se yields higher activation in the aforementioned reward- and emotion-related brain areas, even when the delayed option was chosen. That is, would the choice of the later option in today trials be accompanied with higher activation in these areas compared to the choice of the later option in delay trials? We contrasted today trials in which the later option was chosen with delay trials in which the later option was chosen and found higher activation in the MOFC, MPFC, pACC, PCC, Pcu (see Figure 4C, cf. supporting information file S1, Table S5).

## Discussion

In this fMRI study, participants could not actively choose between different rewards but merely observed intertemporal decisions being made by someone else, either for the participants themselves or for an unknown person. We were interested primarily in the neural activation differences between choices including an immediate reward option (today trials) and choices exclusively including delayed reward options (delay trials) when choices were not made but observed. It was crucial that the beneficiary of the action differed, in that intertemporal decisions could be made for the person who observed the choices (SELF) or for another, entirely unknown person (OTHER).

In summary, the results are consistent with our hypothesis that there is stronger activation in reward- and emotion-related areas for today trials than for delay trials, even when participants merely observe intertemporal choices being made for them by another person. Furthermore, we identified the brain areas involved when the beneficiary of these intertemporal choices made by the decision maker was actually an unknown third party. We will discuss these findings in the context of neuroscientific evidence on intertemporal choice in the following.

### Activations when immediate gratification was possible for SELF

The ventral striatum, MOFC, pACC, MPFC, and vPCC were significantly more involved when one of the two choice options was payable “today,” and when choices were observed being made for SELF. These areas were suggested to belong to a network of brain areas associated with immediate gratification that was reliably shown to be activated in intertemporal decisions, both when individuals chose the sooner option as well as when they were merely presented with the option of a choice between a sooner reward and a later one [e.g. 4,10].

The ventral striatum, MOFC, and pACC were found to be more highly activated in intertemporal choice when an immediate option was available [4,10,17,39], which is also borne out in our findings. These areas are all parts of the rostral limbic system [40]. They have also been reported to be involved in the anticipation and reception of reward in other tasks. For example, the ventral striatum was found to be sensitive to reward size and the probability of receiving an expected reward [41,42,43,44,45]. The pACC, located anterior to the genu of the corpus callosum, was reported to process positive emotions as well as large gains in gambles [46,47]. One of the network components, the MPFC, has been suggested to subserve processes of self-related judgment and attention in studies comparing evaluations of the self and other persons [37,48,49,50,51]. The ventral posterior part of the cingulate cortex was found to be similarly engaged in self-reflection processes [52] and the processing of emotional stimuli [53,54]. In the case of immediate gratification, we conclude by reverse inference that participants were more self-focused and engaged with their feelings toward immediate rewards than toward delayed rewards or rewards for other individuals.

These findings suggest that taken together, these five areas form a network that processes reward- and emotion-related stimuli associated with the self. This network accordingly showed a higher activation when there was a possibility for immediate gratification (i.e., receiving an immediate reward). As in the findings from our previous study [17], this activation was stronger when immediate gratification pertained to the self than when it pertained to a stranger. We assume that the immediacy engenders impatience for the rewards, enhancing reward-related processes when immediate gratification for the self is possible. The prospect of future rewards might not trigger these processes as strongly, since individuals discount future rewards relative to immediate ones [1,2,3]. In contrast, immediacy does not seem to engender impatience for a monetary reward when another person is the beneficiary, which suggests that immediacy concerning rewards for another is not processed by the same network of brain areas as the immediacy concerning rewards for the self is. We discuss the brain areas involved in intertemporal choice for others below.

With our study, we can show that the effects reported for active intertemporal decision making [10,17] are robust *whoever* the decision maker is, but with the important distinction that *I* am the beneficiary of the reward. The mere possibility of obtaining a reward triggers the activation, even when it is not in the control of the individual to actually choose the immediate reward. This finding concurs with a recent study by Levy, et al. [22], which showed that the ventral striatum and MPFC are engaged in the valuation of products *even in the absence of choice*. In that study, various products were presented to individuals situated in the fMRI-scanner. The scans showed that activation in the ventral striatum and MPFC while participants were looking at the different products predicted product choices that were made by participants after they were released from the scanner.

Our results are further supported by a study from Jimura, Chushak and Braver [55], who used primary rewards (liquids) that were available after a post-choice period of 30 or 60 seconds. They reported activation in the vMPFC and ventral striatum to reflect subjective value not just during choice, but in the post-choice period in which participants were waiting to receive the reward as well. This suggests, as the present study does, that not only choice but also reward expectancy plays a role in the evaluation of rewards.

### Activations when immediate gratification was possible for SELF and OTHER

While investigating the brain activation differences between today and delay trials, we found differences in activation that was higher in SELF compared to OTHER trials, but we also found differences in activation that could be observed in both conditions: activation in the dACC, PCC, and Pcu was higher in today trials than in delay trials when participants observed intertemporal choices, whoever benefitted from those choices.

However, since we did not have a hypothesis about activation differences that could be observed in both conditions, we could only speculate here about the mechanisms that are involved irrespective of who the beneficiary is. Summarizing, the present results indicate that the activations found in several studies on active intertemporal decision making are also present when the same decisions are merely observed by the participants. This supports the assumption that a robust brain network is engaged in immediate gratification. The results additionally suggest that certain brain areas are activated irrespective of who the beneficiary of a decision is. This speaks for a more general role of immediacy, which not only pertains to immediate gratification of one’s own needs but to immediacy per se (in contrast to delay). Certainly, this finding has to be investigated more systematically in future studies.

### Activations when immediate or delayed gratification was chosen for SELF

We observed the aforementioned activation differences in reward- and emotion-related brain areas when the choice options for immediate and delayed gratification were presented. In an explorative analysis, we further investigated whether activation in these brain areas was also increased when an immediate reward was actually chosen. Our results suggest that this is the case: Activation in the vStr, MOFC, MPFC, pACC, PCC, and Pcu were higher when a sooner (i.e., immediate) reward in today trials compared to a sooner reward in delay trials was chosen.

However, so far we contrasted choices in today trials with choices in delay trials only. Hence, the activation differences we found could still be due to the mere presence of an immediate reward and not due to the actual choice or availability of this reward. To investigate this open question, we contrasted choices of the immediate with choices of the delayed option *within* today trials. Here, an immediate option was present irrespectively of whether the immediate or the delayed reward was chosen and hence activation differences would be caused by the actual choice, and not by the available options. We found higher activations in the vStr, MOFC, and MPFC for choices of the immediate compared to the delayed option in today trials. In line with previous research, this suggests that not only the *availability* of an immediate reward, but also the *reception* of an immediate reward triggers activation in these brain areas commonly associated with the processing of [41,42,43,44,45]. However, we found no increased activation in the pACC, PCC, and Pcu within today trials when the immediate reward was chosen. This suggests that these areas respond to the mere presence or availability of immediate rewards, but not to their actual reception. To further investigate to what extent the mere presence of immediate gratification drives the reported activation, we contrasted today trials in which the later option was chosen with delay trials in which the later option was chosen. To this end, we compared trials in which immediate gratification was present and trials without it being present, *after* it was determined that the immediate reward was not chosen. Thus we could compare brain activation in today and delay trials without actual immediate gratification. Interestingly, activation in the MOFC, MPFC, pACC, PCC, and Pcu, but not in the vStr, were also increased when the later (i.e., not immediate) option in today trials – in contrast to the later option in delay trials – was chosen. This suggests that the mere presence of immediate gratification triggers a higher activation in these areas, even after participants learned that not the immediate, but a delayed, reward was chosen for them. This is interesting as it suggests a further distinction between the reported brain areas: The fact that the vStr showed increased activation in all but this contrast suggests that this region might play a role in coding the reception of an immediate reward as well as the availability of an immediate reward, but – in contrast to the MPFC and MOFC -- not additionally the mere presence of an immediate reward. Further, since the pACC and medial posterior regions (PCC and Pcu) did not show higher activation when an immediate compared to a delayed reward was chosen in today trials, these regions seem not to be sensitive to the actual reception of an immediate reward, but rather respond to the mere presence of it.

This suggests a division of brain areas into three classes: Those that code the presence and reception of immediate rewards (i.e., the MPFC and MOFC), those that code the availability and reception (i.e., the vStr), and those that only code the mere presence of immediate rewards (i.e., the pACC, PCC, and Pcu).

Again, we have to mention that the interpretation of these effects based on the feedback phase must be taken with caution and it only points towards intriguing results that need to be investigated in future research. We would be pleased to see future studies focusing on the difference between reward presentation and reward reception as well as between one’s own preferences and others’ choices. While the present study gives feedback about the decision maker’s choices, we did not measure whether the participant agreed or disagreed with particular choices. We chose not to in order to keep the participant’s self-perceived influence on the outcome of the choices as low as possible, an important factor to consider. In future studies, however, brain correlates of agreements and disagreements with the other’s choice could be investigated as a way to disentangle processes active during good news (i.e., the feedback that the preferred option was chosen) or bad news (i.e., the feedback that the non-preferred option was chosen).

## Supporting Information

File S1 **Includes the Instructions and Tables S1 to S5.**
(DOCX)
