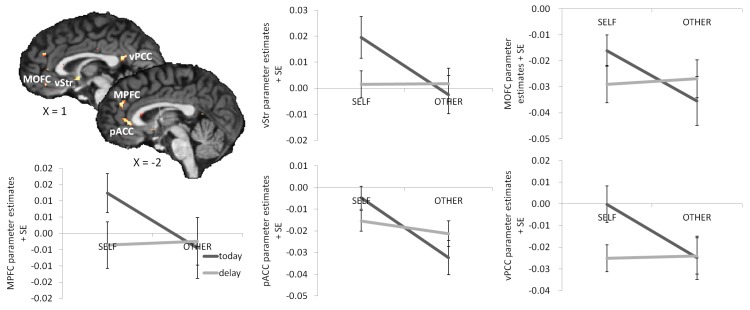# Correction: What Do I Want and When Do I Want It: Brain Correlates of Decisions Made for Self and Other

**DOI:** 10.1371/annotation/4a0ce951-49b2-4533-9cf0-773b5aeabb41

**Published:** 2013-09-13

**Authors:** Konstanze Albrecht, Kirsten G. Volz, Matthias Sutter, D. Yves von Cramon

The correct versions of Figures 2 and 3 are available below.

Figure 2: 

**Figure:**
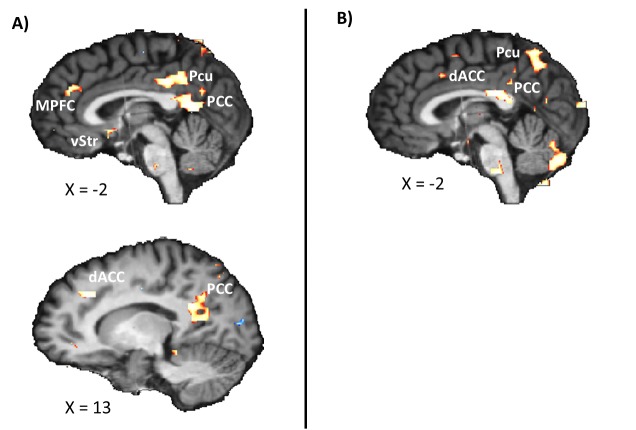


Figure 3: 

**Figure:**