# Supplementary material for: What Do I Want and When Do I Want It: Brain Correlates of Decisions Made for Self and Other
Source: PLoS One. 2013 Aug 22;8(8):e73531. doi: 10.1371/journal.pone.0073531 (PMC3749998; doi:10.1371/journal.pone.0073531)
Supplement: File S1 — Includes the Instructions and Tables S1 to S5. (DOCX) [file pone.0073531.s001.docx]

Supporting Information

*Instructions (translation from the German)*

-------------- INSTRUCTIONS, VERSION #1 (Choices for SELF are observed first) ------------

Dear Participant,

Today’s experiment consists of two parts. Below, you can find the instructions for the first part. After finishing the first part, you will receive the instructions for the second part (on the screen inside the scanner).

**Part 1**

Decisions between two monetary rewards at different points in time

In this part, you will repeatedly see two monetary rewards, and another participant will decide which one of the two rewards you will receive. Specific to each decision is that every reward is associated with a point in time at which you will receive that reward. There are four possible points in time for payment: (a) today, (b) in two weeks, (c) in four weeks, or (d) in six weeks. If the reward with the time “today” is chosen for you, you will receive the money immediately after the experiment. If a reward with the time (b), (c), or (d) is chosen, you will receive the money at that point in time.

40 decisions

In total, 40 decisions will be made for you in this first part. This means that there will be 40 decisions between 2 monetary rewards at 2 different points in time. At the end of the experiment (i.e., after part 2), 1 decision (out of the 40) will be drawn and you will receive the chosen reward at the indicated point in time. (By agreement with you, the reward will be delivered to your house or handed to you in person at an arranged meeting point.)

Please note that every decision is important because after the experiment, one of the decisions will be drawn randomly and paid to you.

After the experiment, you will also be asked to evaluate the decisions of the other person. We would like to know whether you liked his/her decisions.

Procedure on the screen

The exact procedure goes like this:

At first, a fixation cross will be presented, and then the decision options.

Both options are initially marked with yellow triangles.

€ 5.87

today

€ 7.32

in 2 weeks

Presentation of the decision

As soon as the other participant has chosen one of the two rewards for you, there will be a short pause, after which the decision will be presented: The relevant triangle changes to red.

€ 5.87

today

€ 7.32

in 2 weeks

Afterwards, there will be a pause before the next decision options are presented.

Please note that you are the observer of the described procedure! Do NOT press any keys during this time if possible.

Entry of own decisions

However, in some trials we will ask you to make a decision for you YOURSELF between the two rewards (and times)! This will be the case whenever the rewards and times are EQUAL to each other. For example, it could look like this:

€ 2.00

in 2 weeks

€ 3.00

in 3 weeks

or this:

€ 0.00

today

€ 6.00

in 6 weeks

With the two buttons, you either choose the left or the right decision option. You will have enough time to do this, since this is not (!) a reaction time experiment!!!

But these cases are “non-competitive,” i.e., none of these choices will actually be paid out.

Only one of the decisions that the other participant makes for you will actually be drawn and paid to you at the indicated time. Please also be aware that the other participant will not know which alternative you choose in the cases where you may choose yourself. He/she will always make an unbiased decision without knowing your preferences.

Examples

Below are two examples. Please select one of the two options in each of them and mark the option you would have chosen with a cross on the paper.

Example 1:

€ 10.98

today

€ 10.98

in 2 weeks

Example 2:

€ 0.98

today

€ 14.80

in 2 weeks

If you have any questions, please ask them now.

------------ INSTRUCTIONS VERSION #2 (Choices for OTHER are observed first) -----------

Dear Participant!

Today’s experiment consists of two parts. Below you can find the instructions to the first part. Only after finishing the first part will you receive the instructions for the second part (on the screen inside the scanner).

**Part 1**

Decisions between two monetary rewards at different points in time

In this part, you will repeatedly see two monetary rewards, and another participant (P1) will decide which of the two rewards a third participant (P2) will receive. P2, the participant who receives the money, is a participant of a later experiment session. For your task of observing the decisions made for P2, you will receive a flat amount of €5.

Special to each decision is that every reward is associated with a point in time at which P2 will receive the indicated reward. There are four possible points in time for payment: (a) today, (b) in two weeks, (c) in four weeks, or (d) in six weeks. If the reward with the time “today” is chosen for P2, he/she will receive the money immediately after the experiment. If a reward with time (b), (c), or (d) is chosen, P2 will receive the money at the indicated point in time.

Forty decisions

In total, 40 decisions will be made for you in this first part. This means that there will be 40 decisions between 2 monetary rewards at 2 different points in time. At the end of the experiment (i.e., after part 2), one decision (out of the 40) will be drawn and P2 will receive the chosen reward at the indicated point in time. (In agreement with them, the reward will be delivered to their house or handed to them in person at an arranged meeting place.)

Please know that every decision is important, because after the experiment, one of the decisions will be drawn randomly and paid to P2.

After the experiment, you will also be asked to evaluate the decisions of the other person (P1). We would like to know whether you liked his/her decisions.

Procedure on the screen

The exact procedure goes like this:

At first, a fixation cross will be presented, and then the decision options.

Both options are initially marked with yellow triangles.

€ 5.87

today

€ 7.32

in 2 weeks

Presentation of the decision

As soon as P1 chooses one of the two rewards for P2, there will be a short pause, after which the decision will be presented to you: The color of the indicated triangle changes to red.

€ 5.87

today

€ 7.32

in 2 weeks

Afterwards, there will be pause before the next decision options are presented.

Please be aware that you are the observer of the described procedure! Please do NOT press any keys during this time if possible.

Entry of own decisions

However, in some trials we will ask you to decide between the two rewards (and times) for P2 YOURSELF! This will be the case whenever the rewards and times are EQUAL to each other. For example, it could look like this:

€ 2.00

in 2 weeks

€ 3.00

in 3 weeks

or this:

€ 0.00

today

€ 6.00

in 6 weeks

With one of the two buttons, you choose either the left or right decision option. Note that you will have enough time - this is not (!) a reaction time experiment!!!

But, these cases are “non-competitive,” i.e., none of these decisions will actually be paid out.

Only one of the decisions the other participant (P1) makes will be drawn and paid to the other participant (P2) at the indicated time. Note that P1 will not know which alternative you choose in the cases in which you may choose yourself. He/she will always decide in a completely unbiased manner and without knowing your preferences.

Examples

Below are two examples. Please select one of the two options in each of them and mark the option you would have chosen with a cross on the paper.

Example 1:

€ 10.98

today

€ 10.98

in 2 weeks

Example 2:

€ 0.98

today

€ 14.80

in 2 weeks

If you have any questions, please ask them now.

*Table S1.* Set 1 (presented to each participant in random order – for half of the participants this set was used in part 1, for the other half in part 2). The later reward r1’ was calculated by adding x% to the sooner reward r1. Delivery times were t and t’ for r1 and r1’, respectively. The rewards were presented for 2584 to 3389 ms (RT), until a choice was indicated (1=the sooner reward was chosen, 2=the later reward was chosen).

| Experimental trials (not in order of presentation) | | | | | | |
| --- | --- | --- | --- | --- | --- | --- |
| r1 | r1’ | t | t’ | x | RT | choice |
| 8.93 | 9.02 | today | in 2 weeks | 1 | 2584 | 1 |
| 32.2 | 32.52 | today | in 4 weeks | 1 | 2584 | 2 |
| 10.1 | 10.4 | today | in 2 weeks | 3 | 2699 | 2 |
| 34.46 | 35.49 | today | in 4 weeks | 3 | 2699 | 1 |
| 14.03 | 14.73 | today | in 2 weeks | 5 | 2754 | 2 |
| 11.43 | 12 | today | in 4 weeks | 5 | 2754 | 1 |
| 16.03 | 17.63 | today | in 2 weeks | 10 | 2894 | 1 |
| 8.29 | 9.12 | today | in 4 weeks | 10 | 2894 | 2 |
| 28.38 | 32.64 | today | in 2 weeks | 15 | 3319 | 1 |
| 10.9 | 12.54 | today | in 4 weeks | 15 | 3319 | 2 |
| 22.63 | 28.29 | today | in 2 weeks | 25 | 3348 | 2 |
| 13.83 | 17.29 | today | in 4 weeks | 25 | 3348 | 1 |
| 24.37 | 32.9 | today | in 2 weeks | 35 | 2986 | 1 |
| 24.63 | 33.25 | today | in 4 weeks | 35 | 2986 | 2 |
| 6.59 | 9.89 | today | in 2 weeks | 50 | 2670 | 2 |
| 26.47 | 39.71 | today | in 4 weeks | 50 | 2670 | 1 |
| 5.15 | 5.2 | in 2 weeks | in 4 weeks | 1 | 3011 | 1 |
| 31.1 | 31.41 | in 2 weeks | in 6 weeks | 1 | 3011 | 1 |
| 14.72 | 14.87 | in 4 weeks | in 6 weeks | 1 | 3011 | 2 |
| 26.38 | 27.17 | in 2 weeks | in 4 weeks | 3 | 3179 | 1 |
| 29.04 | 29.91 | in 2 weeks | in 6 weeks | 3 | 3179 | 2 |
| 11.57 | 11.92 | in 4 weeks | in 6 weeks | 3 | 3179 | 1 |
| 10.68 | 11.21 | in 2 weeks | in 4 weeks | 5 | 3056 | 2 |
| 30.4 | 31.92 | in 2 weeks | in 6 weeks | 5 | 3056 | 1 |
| 11.3 | 11.87 | in 4 weeks | in 6 weeks | 5 | 3056 | 1 |
| 13.72 | 15.09 | in 2 weeks | in 4 weeks | 10 | 3222 | 1 |
| 22.45 | 24.7 | in 2 weeks | in 6 weeks | 10 | 3222 | 2 |
| 23.6 | 25.96 | in 4 weeks | in 6 weeks | 10 | 3222 | 2 |
| 11.07 | 12.73 | in 2 weeks | in 4 weeks | 15 | 3380 | 2 |
| 33.71 | 38.77 | in 2 weeks | in 6 weeks | 15 | 3380 | 1 |
| 27.49 | 31.61 | in 4 weeks | in 6 weeks | 15 | 3380 | 2 |
| 10.67 | 13.34 | in 2 weeks | in 4 weeks | 25 | 3389 | 2 |
| 27.06 | 33.83 | in 2 weeks | in 6 weeks | 25 | 3389 | 2 |
| 6.75 | 8.44 | in 4 weeks | in 6 weeks | 25 | 3389 | 1 |
| 30.21 | 40.78 | in 2 weeks | in 4 weeks | 35 | 3054 | 1 |
| 33.15 | 44.75 | in 2 weeks | in 6 weeks | 35 | 3054 | 2 |
| 12.25 | 16.54 | in 4 weeks | in 6 weeks | 35 | 3054 | 2 |
| 22.67 | 34.01 | in 2 weeks | in 4 weeks | 50 | 2899 | 2 |
| 10.13 | 15.2 | in 2 weeks | in 6 weeks | 50 | 2899 | 1 |
| 34.06 | 51.09 | in 4 weeks | in 6 weeks | 50 | 2899 | 2 |
| Intermixed catch trials: | | | | | | |
| r1 | r1’ | t | t’ |  | | |
| 0.00 | 2.00 | today | in 2 weeks |  | | |
| 1.00 | 4.00 | in 1 week | in 4 weeks |  | | |
| 1.00 | 5.00 | in 1 week | in 5 weeks |  | | |
| 1.00 | 6.00 | in 1 week | in 6 weeks |  | | |
| 2.00 | 4.00 | in 2 weeks | in 4 weeks |  | | |
| 2.00 | 5.00 | in 2 weeks | in 5 weeks |  | | |
| 2.00 | 6.00 | in 2 weeks | in 6 weeks |  | | |
| 3.00 | 5.00 | in 3 weeks | in 5 weeks |  | | |
| 3.00 | 6.00 | in 3 weeks | in 6 weeks |  | | |
| 4.00 | 6.00 | in 4 weeks | in 6 weeks |  | | |

*Table S2.* Set 2 (presented to each participant in random order – for half the participants this set was used in part 1, for the other half in part 2). The later reward r2’ was calculated by adding x% to the sooner reward r2. Delivery times were t and t’ for r2 and r2’, respectively. The rewards were presented for 2584 to 3389 ms (RT), until a choice was indicated (1=the sooner reward was chosen, 2=the later reward was chosen).

| Experimental trials (not in order of presentation) | | | | | | |  |
| --- | --- | --- | --- | --- | --- | --- | --- |
| r2 | r2’ | t | t’ | x | RT | choice |  |
| 30.62 | 30.93 | today | in 2 weeks | 1 | 2584 | 1 |  |
| 8.14 | 8.22 | today | in 4 weeks | 1 | 2584 | 2 |  |
| 14.19 | 14.62 | today | in 2 weeks | 3 | 2699 | 2 |  |
| 25.26 | 26.02 | today | in 4 weeks | 3 | 2699 | 1 |  |
| 12.41 | 13.03 | today | in 2 weeks | 5 | 2754 | 2 |  |
| 12.78 | 13.42 | today | in 4 weeks | 5 | 2754 | 1 |  |
| 10.16 | 11.18 | today | in 2 weeks | 10 | 2894 | 1 |  |
| 6.14 | 6.75 | today | in 4 weeks | 10 | 2894 | 2 |  |
| 28.37 | 32.63 | today | in 2 weeks | 15 | 3319 | 1 |  |
| 26.5 | 30.48 | today | in 4 weeks | 15 | 3319 | 2 |  |
| 12.67 | 15.84 | today | in 2 weeks | 25 | 3348 | 2 |  |
| 17.82 | 22.28 | today | in 4 weeks | 25 | 3348 | 1 |  |
| 12.69 | 17.13 | today | in 2 weeks | 35 | 2986 | 1 |  |
| 12.28 | 16.58 | today | in 4 weeks | 35 | 2986 | 2 |  |
| 26.92 | 40.38 | today | in 2 weeks | 50 | 2670 | 2 |  |
| 25.45 | 38.18 | today | in 4 weeks | 50 | 2670 | 1 |  |
| 28.57 | 28.86 | in 2 weeks | in 4 weeks | 1 | 3011 | 1 |  |
| 10.07 | 10.17 | in 2 weeks | in 6 weeks | 1 | 3011 | 1 |  |
| 8.73 | 8.82 | in 4 weeks | in 6 weeks | 1 | 3011 | 2 |  |
| 31.99 | 32.95 | in 2 weeks | in 4 weeks | 3 | 3179 | 1 |  |
| 26.26 | 27.05 | in 2 weeks | in 6 weeks | 3 | 3179 | 2 |  |
| 34.18 | 35.21 | in 4 weeks | in 6 weeks | 3 | 3179 | 1 |  |
| 29.05 | 30.5 | in 2 weeks | in 4 weeks | 5 | 3056 | 2 |  |
| 7.49 | 7.86 | in 2 weeks | in 6 weeks | 5 | 3056 | 1 |  |
| 25.66 | 26.94 | in 4 weeks | in 6 weeks | 5 | 3056 | 1 |  |
| 27.36 | 30.1 | in 2 weeks | in 4 weeks | 10 | 3222 | 1 |  |
| 33.41 | 36.75 | in 2 weeks | in 6 weeks | 10 | 3222 | 2 |  |
| 14.17 | 15.59 | in 4 weeks | in 6 weeks | 10 | 3222 | 2 |  |
| 34.09 | 39.2 | in 2 weeks | in 4 weeks | 15 | 3380 | 2 |  |
| 14.64 | 16.84 | in 2 weeks | in 6 weeks | 15 | 3380 | 1 |  |
| 13.7 | 15.76 | in 4 weeks | in 6 weeks | 15 | 3380 | 2 |  |
| 28.97 | 36.21 | in 2 weeks | in 4 weeks | 25 | 3389 | 2 |  |
| 8.42 | 10.53 | in 2 weeks | in 6 weeks | 25 | 3389 | 2 |  |
| 31.13 | 38.91 | in 4 weeks | in 6 weeks | 25 | 3389 | 1 |  |
| 5.95 | 8.03 | in 2 weeks | in 4 weeks | 35 | 3054 | 1 |  |
| 33.45 | 45.16 | in 2 weeks | in 6 weeks | 35 | 3054 | 2 |  |
| 33.57 | 45.32 | in 4 weeks | in 6 weeks | 35 | 3054 | 2 |  |
| 33.18 | 49.77 | in 2 weeks | in 4 weeks | 50 | 2899 | 2 |  |
| 6.32 | 9.48 | in 2 weeks | in 6 weeks | 50 | 2899 | 1 |  |
| 17.06 | 25.59 | in 4 weeks | in 6 weeks | 50 | 2899 | 2 |  |
| Intermixed catch trials: | | | | | | |  |
| r2 | r2’ | t | t’ |  | | |  |
| 0.00 | 2.00 | today | in 2 weeks |  | | |  |
| 1.00 | 4.00 | in 1 week | in 4 weeks |  | | |  |
| 1.00 | 5.00 | in 1 week | in 5 weeks |  | | |  |
| 1.00 | 6.00 | in 1 week | in 6 weeks |  | | |  |
| 2.00 | 4.00 | in 2 weeks | in 4 weeks |  | | |  |
| 2.00 | 5.00 | in 2 weeks | in 5 weeks |  | | |  |
| 2.00 | 6.00 | in 2 weeks | in 6 weeks |  | | |  |
| 3.00 | 5.00 | in 3 weeks | in 5 weeks |  | | |  |
| 3.00 | 6.00 | in 3 weeks | in 6 weeks |  | | |  |
| 4.00 | 6.00 | in 4 weeks | in 6 weeks |  | | |  |

*Table S3.* Catch trials: Mean response time (RT) in seconds

|  | RT | RT following today trials | RT following delay trials |
| --- | --- | --- | --- |
| SELF | 2.42 | 2.67 | 2.30 |
| OTHER | 2.32 | 2.28 | 2.46 |

*Table S4*. Reported are Talairach coordinates of activation peaks in the main contrasts of today trials and delay trials in SELF and OTHER (visualized in Fig. 2), and the interaction contrast (temporal distance x receiver). Only activation blobs that contain a minimum of 10 voxels are reported.

| *Today trials > delay trials SELF* | x | y | z | Max. |
| --- | --- | --- | --- | --- |
| Ventral posterior cingulate cortex | -5 | -54 | 15 | 100.00 |
| Ventral posterior cingulate cortex | 10 | -54 | 15 | 100.00 |
| Dorsal posterior cingulate cortex | -8 | -39 | 36 | 100.00 |
| Dorsal posterior cingulate cortex | 1 | -50 | 36 | 100.00 |
| Dorsal anterior cingulate cortex | 13 | 25 | 33 | 99.99 |
| Medial prefrontal cortex | -2 | 39 | 27 | 100.00 |
| Ventral striatum | 4 | 4 | -3 | 99.99 |
| Medial orbitofrontal cortex | 7 | 34 | -9 | 99.90 |
| Middle temporal gyrus | -44 | -20 | -9 | 100.00 |
| Middle temporal gyrus | 37 | -20 | -18 | 99.98 |
| Intraparietal sulcus | 58 | -50 | 15 | 99.99 |
| Inferior frontal junction | -53 | 25 | 15 | 100.00 |
| Inferior frontal junction | 40 | 13 | 24 | 100.00 |
| Ventral lateral prefrontal cortex | -26 | 52 | -3 | 99.98 |
| Precuneus | 16 | -60 | 36 | 100.00 |
| Precuneus | 11 | -60 | 30 | 100.00 |
| Amygdala | -20 | 1 | -15 | 99.91 |
| Pons | -2 | -29 | -30 | 99.87 |
| Cerebellum | 31 | -47 | -15 | 99.98 |
| Cerebellum | 1 | -56 | -33 | 99.88 |
| Cerebellum | 40 | -59 | -33 | 99.78 |
| *Today trials > delay trials OTHER* | x | y | z | Max |
| Intraparietal sulcus | -56 | -53 | 15 | 100.00 |
| Intraparietal sulcus | 25 | -68 | 48 | 100.00 |
| Inferior frontal junction | -44 | 15 | 21 | 100.00 |
| Inferior frontal junction | 28 | 9 | 21 | 100.00 |
| Ventral lateral prefrontal cortex | -23 | 58 | 9 | 100.00 |
| Precuneus | -15 | -63 | 45 | 100.00 |
| Precuneus | 4 | -42 | 36 | 100.00 |
| dorsal posterior cingulate cortex | 1 | -32 | 27 | 100.00 |
| Anterior dorsolateral prefrontal cortex | 10 | -2 | 39 | 99.72 |
| Superior temporal gyrus | 52 | -8 | 0 | 99.93 |
| Superior occipital gyrus | -29 | -77 | 24 | 99.94 |
| Cuneus | -8 | -65 | 15 | 99.85 |
| Cuneus | -5 | -98 | 9 | 99.99 |
| Cuneus | 10 | -71 | 9 | 99.95 |
| Middle frontal gyrus | 37 | 34 | 18 | 99.96 |
| Thalamus | -8 | -20 | 3 | 99.99 |
| Thalamus | 10 | -8 | 3 | 99.97 |
| Thalamus | -11 | -8 | 0 | 99.54 |
| Hippocampus | -11 | 16 | -6 | 100.00 |
| Middle temporal gyrus | -59 | -2 | -27 | 99.99 |
| Middle temporal gyrus | -35 | -83 | -30 | 99.99 |
| Middle temporal gyrus | -50 | 10 | -30 | 99.96 |
| Middle temporal gyrus | 55 | -62 | 0 | 100.00 |
| Inferior temporal gyrus | -41 | -11 | -33 | 99.95 |
| Caudate head | -11 | 16 | -6 | 99.81 |
| Parahippocampal gyrus | -26 | 16 | -18 | 99.62 |
| Parahippocampal gyrus | -17 | -8 | -21 | 99.85 |
| Cerebellum | -20 | -77 | -45 | 99.76 |
| Cerebellum | 54 | -71 | -45 | 100.00 |
| *Interaction contrast*  *(temporal distance x receiver)* | x | y | z | Max |
| Ventral striatum | 1 | 1 | -3 | 99.81 |
| Medial orbitofrontal cortex | 4 | 37 | -9 | 99.97 |
| Medial prefrontal cortex | -2 | 37 | 27 | 99.92 |
| Pregenual anterior cingulate cortex | -5 | 28 | 3 | 100.00 |
| ventral posterior cingulate cortex | -29 | -14 | 21 | 99.93 |
| dorsal posterior cingulate cortex | 22 | -50 | 21 | 100.00 |
| Temporal parietal junction | -41 | -65 | 18 | 100.00 |
| Precuneus | -11 | -56 | 12 | 99.87 |
| Middle temporal gyrus | -41 | -71 | 30 | 99.90 |
| Middle temporal gyrus | 37 | -62 | 30 | 100.00 |

| *SELF > OTHER (collapsed over today and delay trials)* | x | y | z | Max |
| --- | --- | --- | --- | --- |
| Dorsal anterior cingulate cortex | -8 | 4 | 42 | 99.60 |
| Pregenual anterior cingulate cortex | -2 | 31 | 3 | 99.85 |
| Pregenual anterior cingulate cortex | 1 | 28 | 18 | 99.86 |
| Cuneus | -1 | -53 | 6 | 99.70 |
| Cuneus | -8 | -71 | 21 | 99.96 |
| Cuneus | -17 | -92 | 33 | 100.00 |
| Cuneus | 10 | -62 | 3 | 99.98 |
| Precuneus | -23 | -74 | 39 | 99.80 |
| Precuneus | 10 | -80 | 48 | 99.90 |
| Precuneus | 13 | -56 | 63 | 99.66 |
| Posterior cingulate cortex | 22 | -59 | 9 | 99.87 |
| Precentral gyrus | -38 | -17 | 54 | 99.93 |
| Middle frontal gyrus | 46 | 4 | 45 | 99.89 |
| Superior parietal lobe | -26 | -53 | 60 | 99.84 |
| Inferior parietal lobe | 52 | -35 | 51 | 99.98 |
| Postcentral gyrus | -50 | -23 | 30 | 100.00 |
| Postcentral gyrus | 55 | -23 | 36 | 99.74 |
| Postcentral gyurs | 52 | -17 | 18 | 99.82 |
| Insula cortex | 40 | -17 | 0 | 99.94 |
| Inferior frontal junction | -56 | -2 | 30 | 99.87 |
| Thalamus | -17 | -32 | 0 | 99.86 |
| Inferior temporal gyrus | 49 | -68 | -3 | 99.95 |

*Table S5.* Reported are Talairach coordinates of activation peaks of the ROIs in the response-dependent contrasts of today trials and delay trials in SELF (visualized in Fig. 4). Only activation blobs that contain a minimum of 10 voxels are reported.

| *Today trials in which the sooner reward was chosen > delay trials in which the sooner reward chosen* | x | y | z | Max. |
| --- | --- | --- | --- | --- |
| Ventral Striatum | 10 | 10 | 0 | 100.00 |
| Ventral Striatum | -11 | 10 | 3 | 99.95 |
| Medial orbitofrontal cortex | -8 | 25 | -9 | 99.98 |
|  |  |  |  |  |
| Medial prefrontal cortex | -14 | 52 | 12 | 100.00 |
| Medial prefrontal cortex | -14 | 52 | 21 | 100.00 |
| Pregenual anterior cingulate cortex | 7 | 28 | 18 | 100.00 |
| Posterior cingulate cortex | 4 | -59 | 21 | 100.00 |
| Precuneus | 1 | -44 | 33 | 99.99 |
| Precuneus | -11 | -44 | 33 | 99.97 |
| Precuneus | -5 | -62 | 48 | 99.81 |
| *Today trials in which the sooner reward was chosen > Today trials in whicht the later reward chosen* | x | y | z | Max. |
| Ventral striatum | 7 | 10 | 0 | 99.99 |
| Medial orbitofrontal cortex | -8 | 19 | -21 | 99.83 |
| Medial prefrontal cortex | -5 | 46 | 27 | 99.91 |
| *Today trials in which the later reward was chosen > Delay trials in whicht the later reward chosen* | x | y | z | Max. |
| Medial orbitofrontal cortex | 4 | 19 | -3 | 99.34 |
| Medial prefrontal cortex | 4 | 49 | 15 | 100.00 |
| Medial prefrontal cortex | -2 | 55 | 6 | 99.84 |
| Pregenual anterior cingulate cortex | 13 | 25 | 6 | 100.00 |
| Pregenual anterior cingulate cortex | -2 | 31 | 15 | 100.00 |
| Posterior cingulate cortex | 4 | -53 | 18 | 100.00 |
| Posterior cingulate cortex | -14 | -56 | 12 | 100.00 |
| Precuneus | 1 | -50 | 36 | 99.95 |
| Precuneus | -11 | -56 | 42 | 100.00 |
